# Supplementary material for: Cellular stress promotes NOD1/2‐dependent inflammation via the endogenous metabolite sphingosine‐1‐phosphate
Source: EMBO J. 2021 May 4;40(13):e106272. doi: 10.15252/embj.2020106272 (PMC8246065; doi:10.15252/embj.2020106272)
Supplement: Supplementary file 1 — Appendix [file EMBJ-40-e106272-s001.pdf]

Table of contents

Appendix Figure S1 .... *2*

Appendix Figure S2 .... *3*

Appendix Figure S3 .... *4*

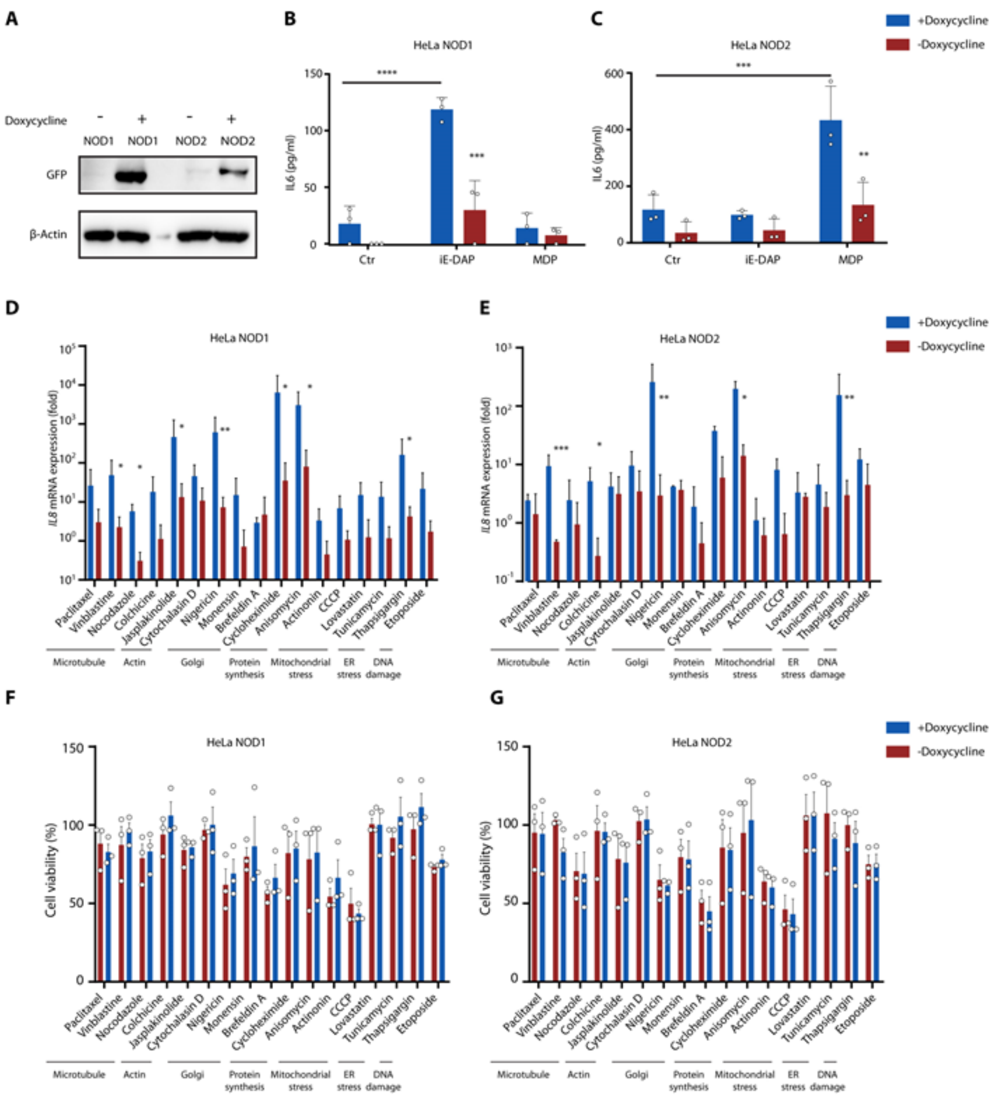

Appendix Figure S1

(**A**) Western blot analysis of NOD1 or NOD2 expression in HeLa inducible NOD1 or NOD2 cells in absence or presence of doxycycline.

(**B, C**) ELISA analysis of IL6 production in supernatants of HeLa NOD1 cells (**B**) or NOD2 cells (**C**) in response to different stimuli.

(**D, E**) qRT-PCR analysis of *IL8* expression upon indicated stimulations without or with NOD1 (**D**) or NOD2 (**E**) expression. Means±SD of one representative experiment from 3 independent experiments.

(**F, G**) Cell viability analysis of HeLa NOD1 or NOD2 cells in absence or presence of doxycycline. (**B, C, F, G**) Means±SEM of 3 independent experiments. Each dot represents one independent experiment. P values were calculated using two-way ANOVA. (**) $p \leq 0.01$, (***) $p \leq 0.001$.

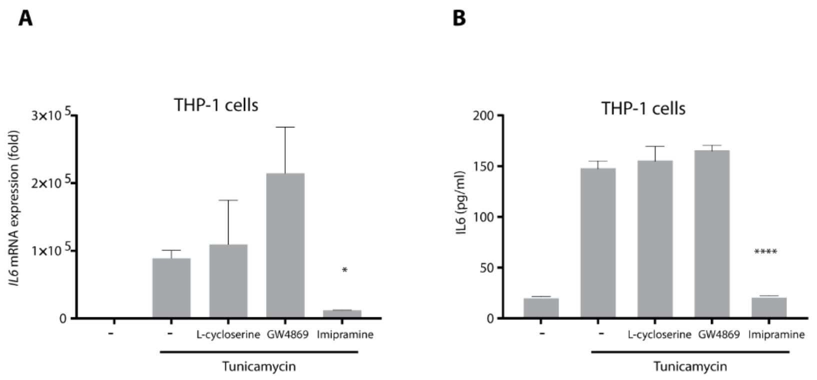

Appendix Figure S2

(**A, B**) qRT-PCR analysis of IL6 expression (**A**) and ELISA analysis of IL6 production (**B**) in THP-1 cell pretreated with various inhibitors against sphingolipid metabolism upon Tunicamycin (5 µg/ml) stimulation. Means±SD of one representative experiment out of 3 independent experiments. P values were calculated using Student's t test (**A**) or one-way ANOVA (**B**). (*) p ≤0.05, (**) p ≤ 0.01, (***) p ≤ 0.001, (****) p ≤ 0.0001.

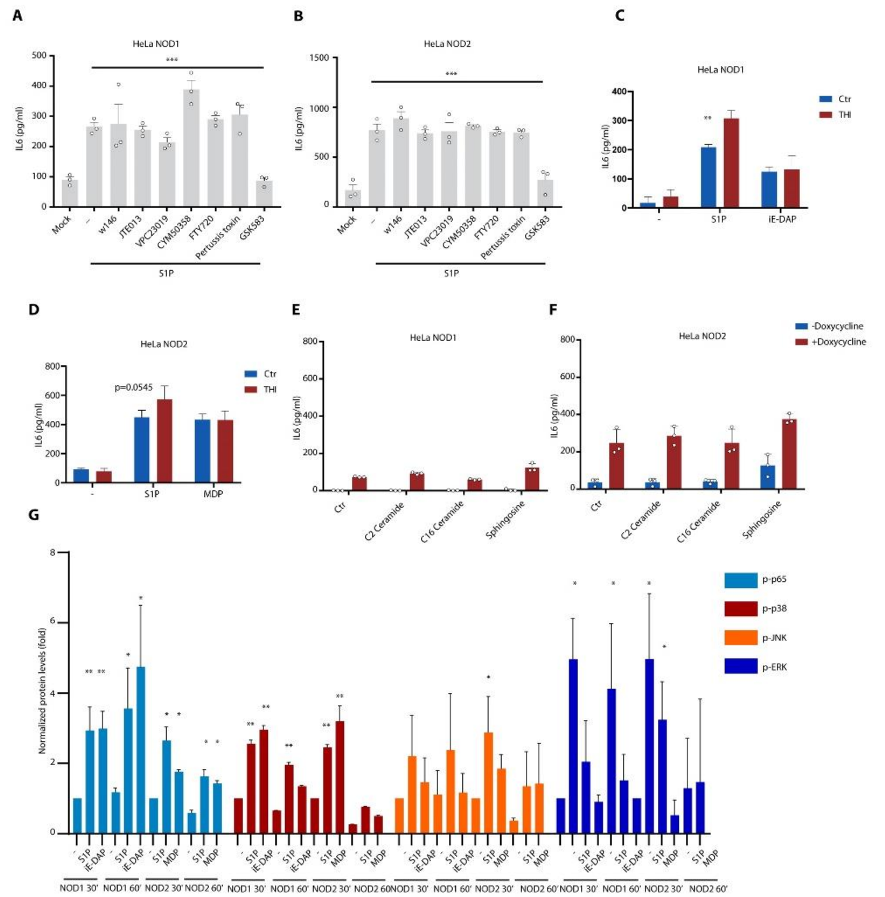

Appendix Figure S3

(**A, B**) ELISA analysis of IL6 in supernatants of HeLa NOD1 (A) or NOD2 (B) cells in the presence of inhibitors of S1PRs upon various stimulations. After doxycycline induction overnight, HeLa NOD1 or NOD2 cells were pretreated with W146 (10 µM), JTE013 (2 µM), VPC23019 (10 µM), CYM50358 (10 µM), FTY720 (10 µM), Pertussis toxin (200 ng/ml) or GSK583 (25 µM) for 30 min and then stimulated with S1P (10 µM) in the presence of digitonin (5 µg/ml) overnight.

(**C, D**) ELISA analysis of IL6 in supernatants of HeLa NOD1 (**C**) or NOD2 (**D**) cells stimulated with S1P (10 µM), iE-DAP (20 µM) or MDP (20 µM) in the presence or absence of THI (50 µM).

(**E, F**) ELISA analysis of IL6 in supernatants of HeLa NOD1 (**E**) or NOD2 (**F**) cells stimulated with different classes of lipids. HeLa NOD1 or NOD2 cells were stimulated with C2 Ceramide (10 µM), C16 Ceramide (10 µM) or Sphingosine (10 µM) in absence or presence of doxycycline.

(**G**) Quantification of normalized protein levels of p-p65, p-p38, p-JNK and p-ERK. The band intensity of each protein was measured with ImageJ and normalized to β-Actin. Then relative levels were calculated against corresponding controls (HeLa NOD1 or HeLa NOD2 without treatment at 30'). Means±SD of 3 independent experiments. (**A, B, E, F**) Means±SEM of 3 independent experiments. Each dot represents one independent experiment. (**C, D**) Means±SD of one representative experiment from 3 independent experiments. P values were calculated using two-way ANOVA or one-way ANOVA. (*) p ≤0.05, (**) p ≤ 0.01, (***) p ≤ 0.001, (****) p ≤ 0.0001.
