# Supplementary material for: Cellular stress promotes NOD1/2‐dependent inflammation via the endogenous metabolite sphingosine‐1‐phosphate
Source: EMBO J. 2021 May 4;40(13):e106272. doi: 10.15252/embj.2020106272 (PMC8246065; doi:10.15252/embj.2020106272)
Supplement: Supplementary file 2 — Expanded View Figures PDF [file EMBJ-40-e106272-s003.pdf]

Expanded View Figures

Figure EV1. NOD1/2 activation induced by perturbation of cellular homeostasis is not due to peptidoglycan contaminants in the serum.

A ELISA analysis of IL6 production in supernatants of HeLa inducible GFP cells. HeLa inducible GFP cells were induced in the absence or presence of doxycycline overnight and stimulated with various stimuli for 20 h, and afterward, supernatants were collected for ELISA.

B–E ELISA analysis of IL6 production in supernatants of HeLa inducible NOD1 or NOD2 cells in DMEM full medium or Opti-MEM (B, C) or with Dynasore (50 μM) (D, E).

Data information: Means ± SEM of three independent experiments. Each dot represents one independent experiment. *P* values were calculated using two-way ANOVA. *$P \leq 0.05$, **$P \leq 0.01$, and ***$P \leq 0.001$.

© 2021 The Authors

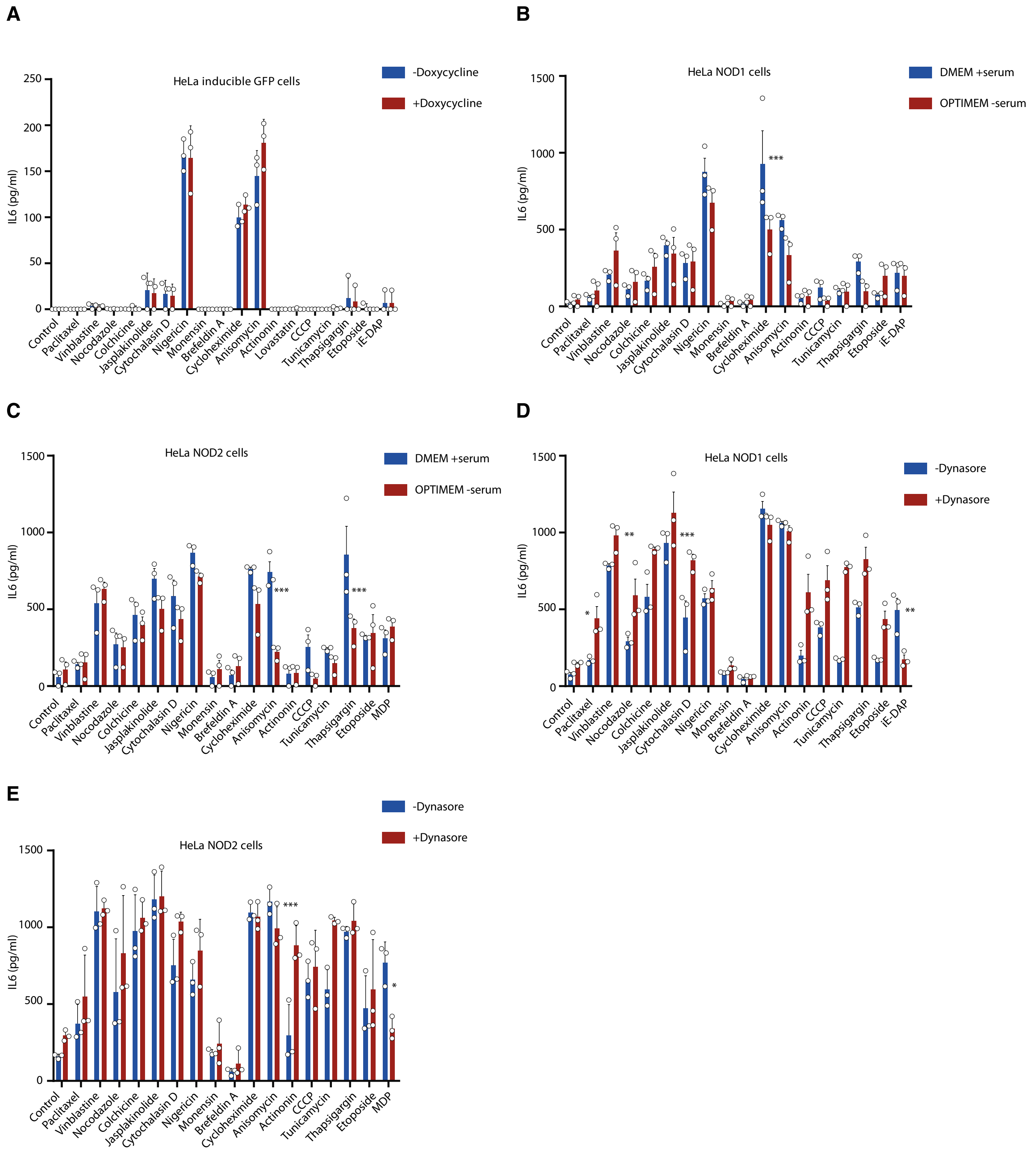
A
HeLa inducible GFP cells
-Doxycycline
+Doxycycline
IL6 (pg/ml)
Control
Paclitaxel
Vinblastine
Nocodazole
Colchicine
Jasplakinolide
Cytochalasin D
Nigericin
Monensin
Brefeldin A
Cycloheximide
Anisomycin
Actinonin
Lovastatin
CCCP
Tunicamycin
Thapsigargin
Etoposide
iE-DAP
B
HeLa NOD1 cells
DMEM +serum
OPTIMEM -serum
IL6 (pg/ml)
***
C
HeLa NOD2 cells
DMEM +serum
OPTIMEM -serum
IL6 (pg/ml)
***
***
MDP
D
HeLa NOD1 cells
-Dynasore
+Dynasore
IL6 (pg/ml)
*
**
***
**
E
HeLa NOD2 cells
-Dynasore
+Dynasore
IL6 (pg/ml)
***
*

Figure EV1.

Figure EV2. NOD1/2 are required for MAPK and NF-κB activation upon ER stress.

A, B Multiplex analysis of CCL2 (A) and IL16 (B) production in supernatants of WT, *Nod1/2* dKO, and *Rip2* KO BMDMs upon indicated stimuli for 20 h.

C Western blot analysis of NF-κB and MAPK activation in BMDMs. WT, *Nod1/2* dKO, and *Rip2* KO BMDMs were stimulated with tunicamycin (5 μM) at different time points, and then, cell lysates were collected for Western blot analysis.

D Quantification of normalized protein levels of p-p65, p-p38, p-JNK, and p-ERK. The band intensity of each protein was measured with ImageJ and normalized to β-actin. Then, relative levels were calculated against corresponding controls (WT, *Nod1/2* dKO, *Rip2* KO without treatment).

Data information: (A, B, D) Means ± SD of three independent experiments. Each dot represents one independent experiment. *P* values were calculated using one-way or two-way ANOVA. $^*P \le 0.05$, $^{**}P \le 0.01$, $^{***}P \le 0.001$, and $^{****}P \le 0.0001$.

Source data are available online for this figure.

 © 2021 The Authors

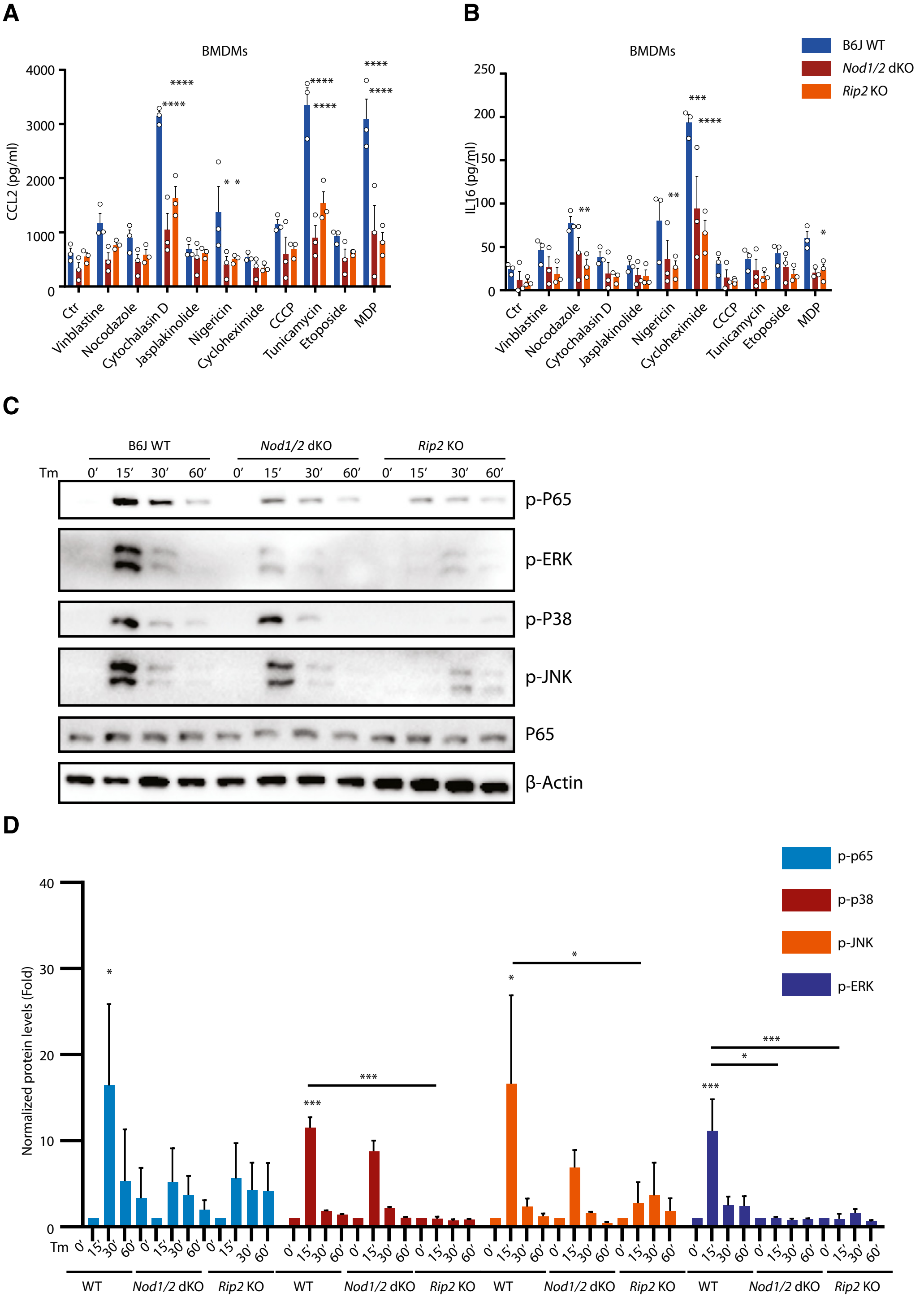
A
BMDMs
CCL2 (pg/ml)
Ctr
Vinblastine
Nocodazole
Cytochalasin D
Jasplakinolide
Nigericin
Cycloheximide
CCCP
Tunicamycin
Etoposide
MDP
B
BMDMs
IL16 (pg/ml)
B6J WT
Nod1/2 dKO
Rip2 KO
C
B6J WT
Nod1/2 dKO
Rip2 KO
Tm
p-P65
p-ERK
p-P38
p-JNK
P65
β-Actin
D
Normalized protein levels (Fold)
p-p65
p-p38
p-JNK
p-ERK
WT
Nod1/2 dKO
Rip2 KO

Figure EV2.

Figure EV3. Sphingosine kinases are critical for IL6 production and MAPK activation upon ER stress.

A The *de novo* and the hydrolysis pathway of sphingolipid metabolism and inhibitors used in this study.

B Lipidomic analysis of the abundance of various lipid classes in cells upon indicated stimulations. Human fibroblasts were treated with cytochalasin D (5 μM), vinblastine (10 μM), cycloheximide (25 μM), CCCP (5 μM), etoposide (5 μM), or tunicamycin (5 μg/ml) for 2 h. Cell lysates were collected for lipidomic profiling. Heatmap shows the mean of 2 independent experiments. CE: cholesteryl ester, Cer: ceramide, CL: cardiolipin, DAG, HexCer: hexosylceramide, LPA: lyso-phosphatidate, LPC: lyso-phosphatidylcholine, LPE: lyso-phosphatidylethanolamine, LPE O-: ether-linked LPE, LPG: lyso-phosphatidylglycerol, LPI: lyso-phosphatidylinositol, PA: phosphatidate, PC: phosphatidylcholine, PC O-: ether-linked PC, PE: phosphatidylethanolamine, PE O-: ether-linked PE, PG: phosphatidylglycerol, PI: phosphatidylinositol, PS: phosphatidylserine, SM: sphingomyelin, and TAG: triacylglycerol.

C–G qRT–PCR analysis of *IL6* expression (C, E) and ELISA analysis of IL6 production (D, F, G) in THP-1 cell (C, D), BMDMs (E, F), or human $CD14^+$ monocytes (G) pretreated with various inhibitors against sphingolipid metabolism upon tunicamycin (5 μg/ml) stimulation.

H Western blot analysis of MAPK activation in B6N WT, *Sphk1* KO, and *Sphk2* KO BMDMs upon tunicamycin stimulation. B6N WT, *Sphk1* KO, and *Sphk2* KO BMDMs were treated with tunicamycin (5 μg/ml) and at different time points, cell lysates were collected for SDS–PAGE.

I Quantification of normalized protein levels of p-p38, p-JNK, and p-ERK. The band intensity of each protein was measured with ImageJ and normalized to β-actin. Then relative levels were calculated against corresponding controls (WT, *Sphk1* KO, *Sphk2* KO without treatment). Means ± SD of three independent experiments.

J Western blot analysis of SPHK1 and SPHK2 in HeLa control (scrambled shRNA) and SPHK1/2 knockdown cells.

Data information: (C-G) Means ± SEM of three independent experiments (C-F) or 4 different donors (G). Each dot represents one independent experiment (C-F) or one donor (G). *P* values (C-G) were calculated using one-way ANOVA. $^*P \leq 0.05$, $^{**}P \leq 0.01$, $^{***}P \leq 0.001$, and $^{****}P \leq 0.0001$.

Source data are available online for this figure.

 © 2021 The Authors

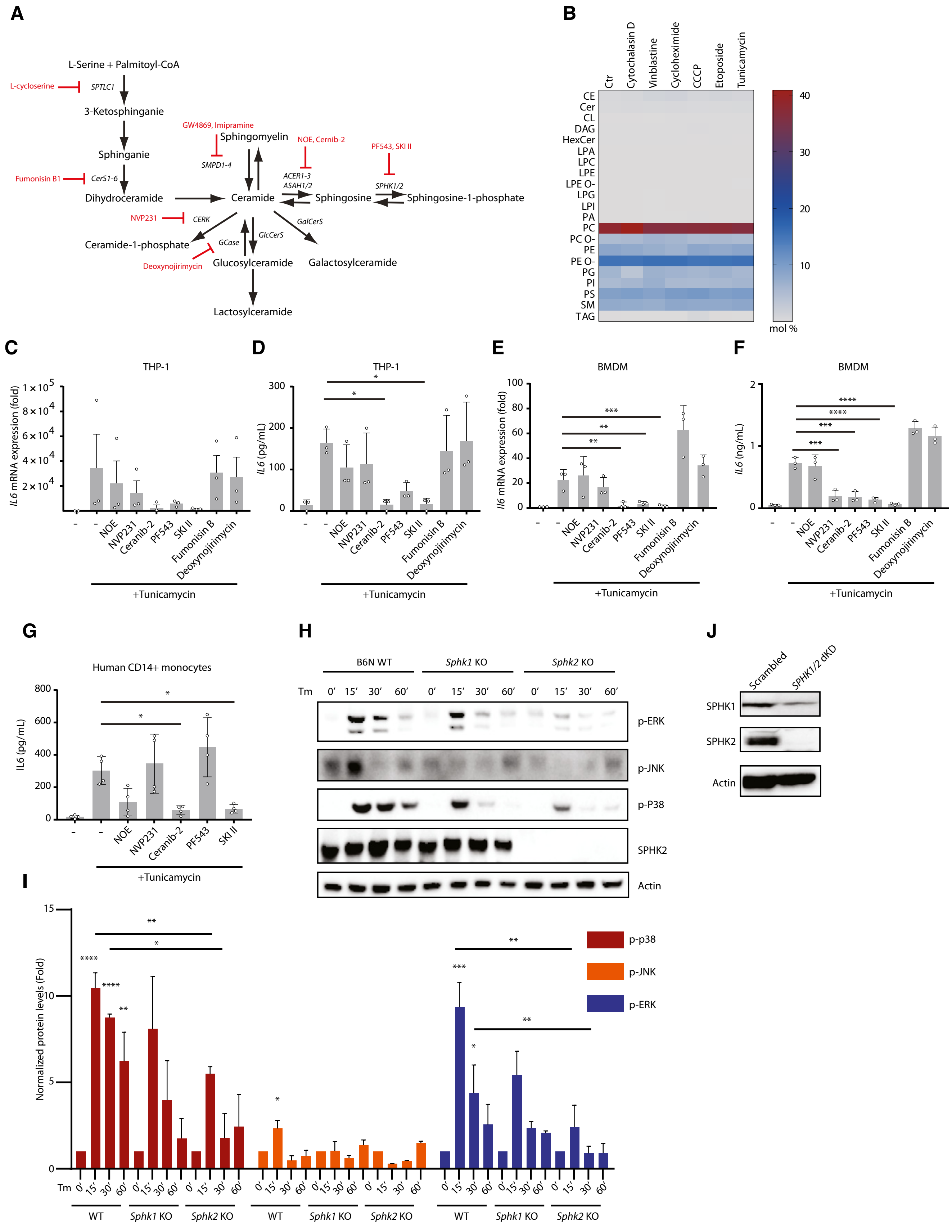
A
L-Serine + Palmitoyl-CoA
L-cycloserine
SPTLC1
3-Ketosphinganie
Sphinganie
Fumonisin B1
CerS1-6
Dihydroceramide
GW4869, Imipramine
Sphingomyelin
SMPD1-4
Ceramide
NOE, Cernib-2
ACER1-3
ASAH1/2
Sphingosine
PF543, SKI II
SPHK1/2
Sphingosine-1-phosphate
NVP231
CERK
Ceramide-1-phosphate
GCase
GlcCerS
GalCerS
Deoxynojirimycin
Glucosylceramide
Galactosylceramide
Lactosylceramide
B
Ctr
Cytochalasin D
Vinblastine
Cycloheximide
CCCP
Etoposide
Tunicamycin
CE
Cer
CL
DAG
HexCer
LPA
LPC
LPE
LPE O-
LPG
LPI
PA
PC
PC O-
PE
PE O-
PG
PI
PS
SM
TAG
mol %
C
THP-1
Il6 mRNA expression (fold)
+Tunicamycin
D
THP-1
IL6 (pg/mL)
E
BMDM
Il6 mRNA expression (fold)
F
BMDM
IL6 (ng/mL)
NOE
NVP231
Ceranib-2
PF543
SKI II
Fumonisin B
Deoxynojirimycin
G
Human CD14+ monocytes
IL6 (pg/mL)
H
B6N WT
Sphk1 KO
Sphk2 KO
Tm
p-ERK
p-JNK
p-P38
SPHK2
Actin
J
Scrambled
SPHK1/2 dKD
SPHK1
SPHK2
Actin
I
Normalized protein levels (Fold)
p-p38
p-JNK
p-ERK
WT
Sphk1 KO
Sphk2 KO

Figure EV3.

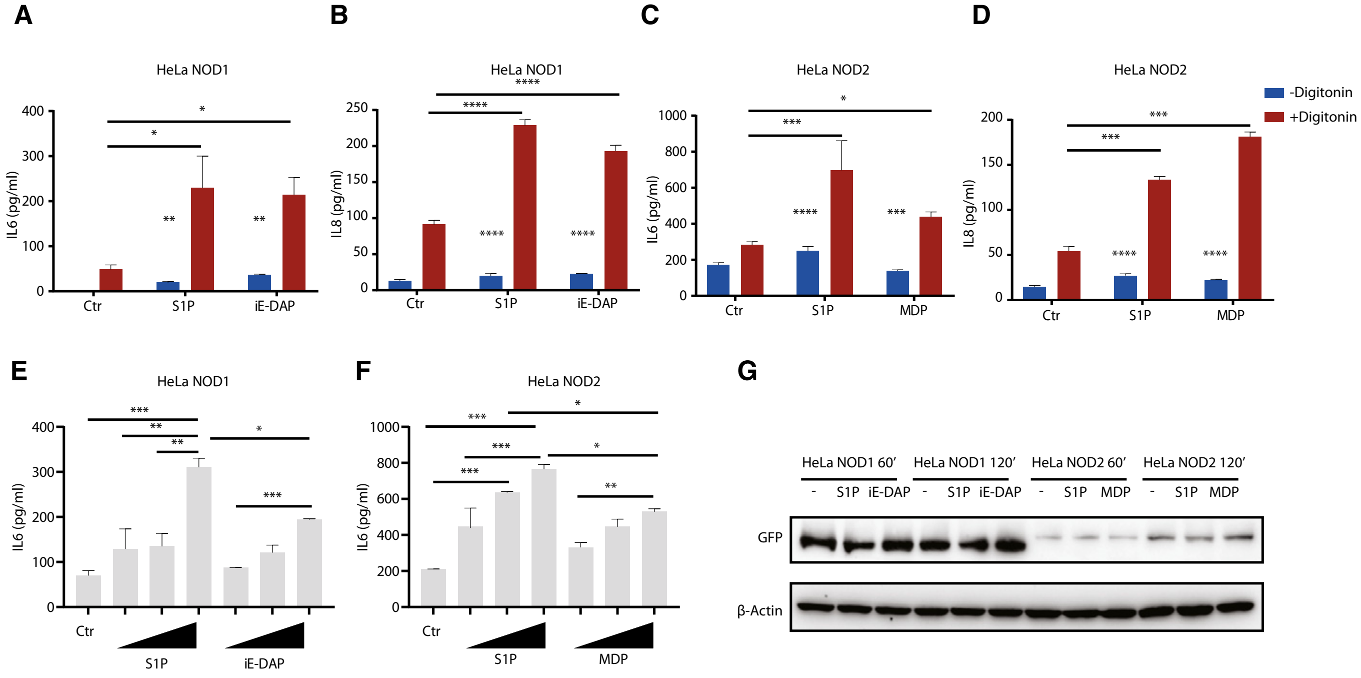

Figure EV4. Cytosolic delivery of S1P induces NOD1/2 activation.

A–D ELISA analysis of IL6 (A, C) or IL8 (B, D) in supernatants of HeLa NOD1 (A, B) or NOD2 cells (C, D) in the absence or presence of digitonin. After doxycycline induction overnight, HeLa NOD1 or NOD2 cells were stimulated with S1P (10 μM), iE-DAP (20 μM), or MDP (20 μM) in the absence or presence of digitonin (5 μg/ml).

E, F ELISA analysis of IL6 in supernatants of HeLa NOD1 (E) or NOD2 cells (F). After doxycycline induction overnight, HeLa NOD1 or NOD2 cells were stimulated with S1P (0.5, 5, or 20 μM), iE-DAP (0.5, 5, or 20 μM), or MDP (0.5, 5, or 20 μM) in the presence of digitonin (5 μg/ml).

G Western blot analysis of NOD1-GFP or NOD2-GFP levels upon stimulation. After doxycycline induction overnight, HeLa NOD1 or NOD2 cells were stimulated with S1P (20 μM), iE-DAP (20 μM), or MDP (20 μM) in the presence of digitonin for indicated time points, and then, cell lysates were collected for Western blot analysis.

Data information: Means ± SEM of three independent experiments. *P* values were calculated using one-way or two-way ANOVA. *$P \leq 0.05$, **$P \leq 0.01$, ***$P \leq 0.001$, and ****$P \leq 0.0001$.

Source data are available online for this figure.

Figure EV5. S1P other than C16 ceramide binds to NOD1 via interacting with histidine at position 517.

A dSTORM imaging of HeLa NOD1 or NOD2 upon S1P stimulation. After stimulation with S1P (20 μM), iE-DAP (50 μM), or MDP (50 μM) for 2 h, HeLa NOD1 or NOD2 cells were fixed, permeabilized, and stained with anti-GFP antibody and Alex Fluor 647-conjugated secondary antibody. Images were reconstructed from 10,000 raw frames. Scale bar: 100 nm.

B–D MST analysis of binding affinity of purified NOD1 (B), NOD2 (C), or cell lysates expressing NOD1 mutants (D) with different ligands.

E, F IL6 ELISA analysis of HeLa NOD1 or NOD2 cells co-stimulated by S1P with iE-DAP or MDP. HeLa NOD1 (E) or NOD2 (F) cells were stimulated with S1P (2 μM), iE-DAP, or MDP (3, 20, 50 μM) or both in the presence of digitonin. After 20-h stimulation, supernatants were collected for ELISA analysis.

G IL8 ELISA analysis of cells expressing NOD2 1007fs upon stimulation. HEK293T cells were transfected with empty vector (mock) or NOD2 1007fs, and then, cells were stimulated with S1P (20 μM) or MDP (20 μM) for 48 h in the presence of digitonin. Means ± SEM of three independent experiments. Each dot represents one independent experiment. *P* values were calculated using two-way ANOVA. *$P \leq 0.05$.

H IL8 ELISA analysis of supernatants from cells expressing NOD1 mutants upon S1P or iE-DAP stimulation. Heatmap showed mean values from 5 independent experiments.

I SDS–PAGE analysis of purified NOD1 WT-GFP and NOD1 H517A-GFP.

 © 2021 The Authors

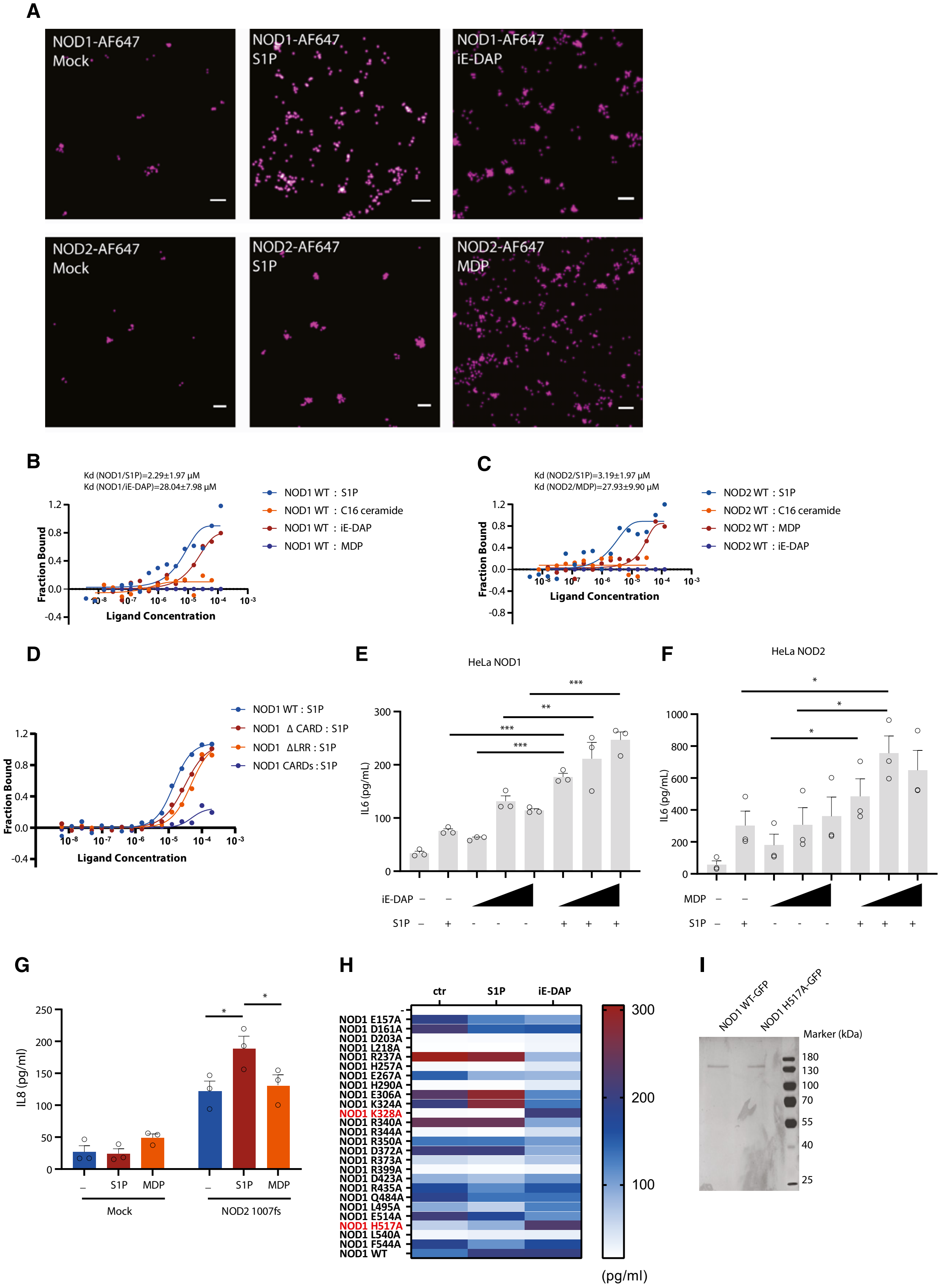
A
NOD1-AF647
Mock
NOD1-AF647
S1P
NOD1-AF647
iE-DAP
NOD2-AF647
Mock
NOD2-AF647
S1P
NOD2-AF647
MDP
B
Kd (NOD1/S1P)=2.29±1.97 μM
Kd (NOD1/iE-DAP)=28.04±7.98 μM
NOD1 WT : S1P
NOD1 WT : C16 ceramide
NOD1 WT : iE-DAP
NOD1 WT : MDP
Fraction Bound
Ligand Concentration
C
Kd (NOD2/S1P)=3.19±1.97 μM
Kd (NOD2/MDP)=27.93±9.90 μM
NOD2 WT : S1P
NOD2 WT : C16 ceramide
NOD2 WT : MDP
NOD2 WT : iE-DAP
Fraction Bound
Ligand Concentration
D
NOD1 WT : S1P
NOD1 Δ CARD : S1P
NOD1 ΔLRR : S1P
NOD1 CARDs : S1P
Fraction Bound
Ligand Concentration
E
HeLa NOD1
IL6 (pg/mL)
iE-DAP
S1P
F
HeLa NOD2
IL6 (pg/mL)
MDP
S1P
G
IL8 (pg/ml)
S1P
MDP
Mock
NOD2 1007fs
H
ctr
S1P
iE-DAP
NOD1 E157A
NOD1 D161A
NOD1 D203A
NOD1 L218A
NOD1 R237A
NOD1 H257A
NOD1 E267A
NOD1 H290A
NOD1 E306A
NOD1 K324A
NOD1 K328A
NOD1 R340A
NOD1 R344A
NOD1 R350A
NOD1 D372A
NOD1 R373A
NOD1 R399A
NOD1 D423A
NOD1 R435A
NOD1 Q484A
NOD1 L495A
NOD1 E514A
NOD1 H517A
NOD1 L540A
NOD1 F544A
NOD1 WT
300
200
100
(pg/ml)
I
NOD1 WT-GFP
NOD1 H517A-GFP
Marker (kDa)
180
130
100
70
55
40
25

Figure EV5.
