# Supplementary figures and images for: Cellular stress promotes NOD1/2‐dependent inflammation via the endogenous metabolite sphingosine‐1‐phosphate
Source: EMBO J. 2021 May 4;40(13):e106272. doi: 10.15252/embj.2020106272 (PMC8246065; doi:10.15252/embj.2020106272)

**Figure EV4G**

**GFP**

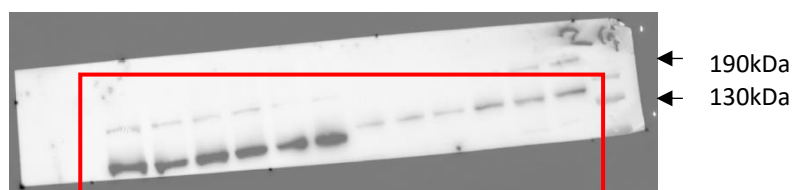

**$\beta$ -Actin**

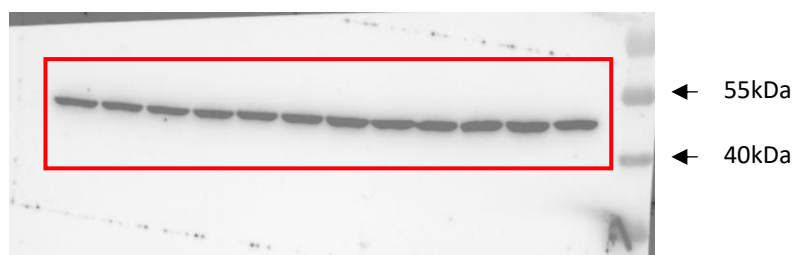

Supplement: Supplementary file 3 — Source Data for Expanded View and Appendix [file EMBJ-40-e106272-s006.zip › EV_and_Appendix_Source_Data/EMBOJ-2020-106272R1-Figure_EV4G_Source_Data-sd.pdf]

## Appendix Figure S1

### GFP

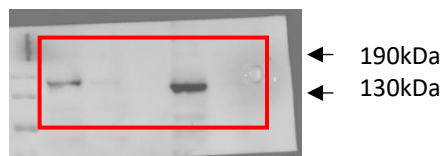

### $\beta$ -Actin

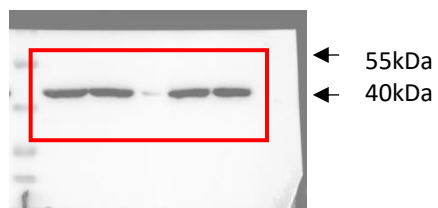

Supplement: Supplementary file 3 — Source Data for Expanded View and Appendix [file EMBJ-40-e106272-s006.zip › EV_and_Appendix_Source_Data/EMBOJ-2020-106272R1-Appendix_Figure_S1_Source_Data-sd.pdf]

**Figure EV3H**

**P-ERK (Upper) and P-P38 (Lower)**

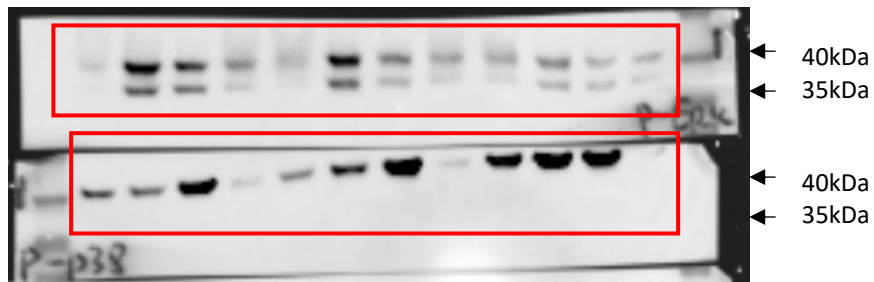

**P-JNK**

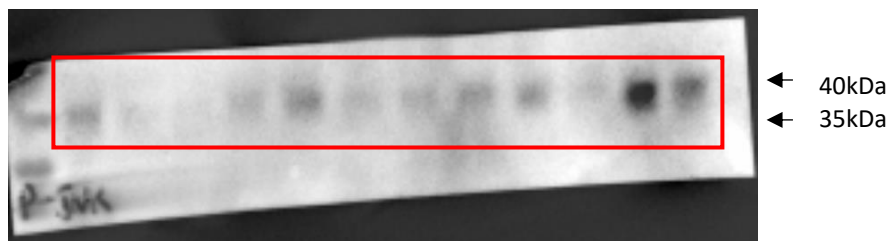

**SPHK2**

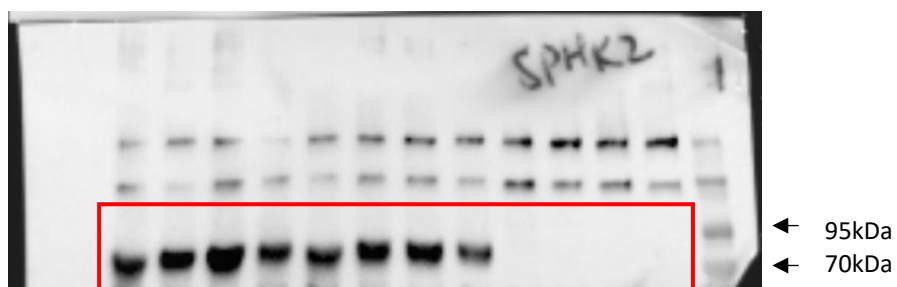

**$\beta$ -Actin**

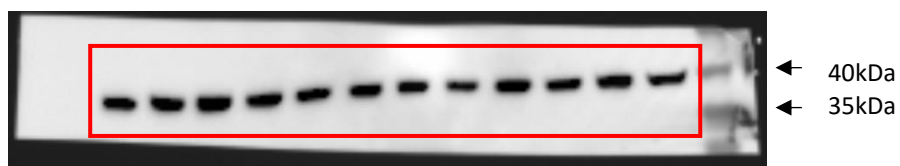

**Figure EV3J**

**SPHK1**

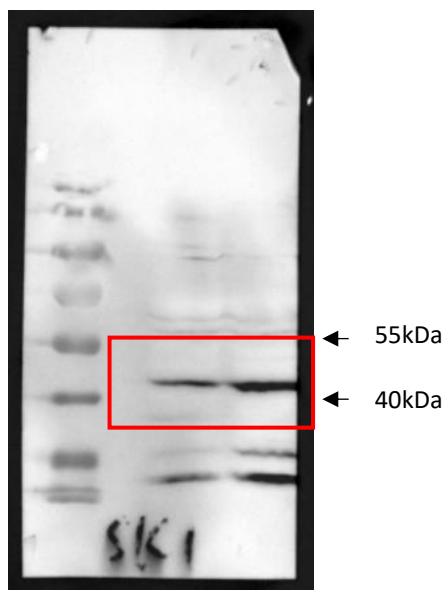

**SPHK2**

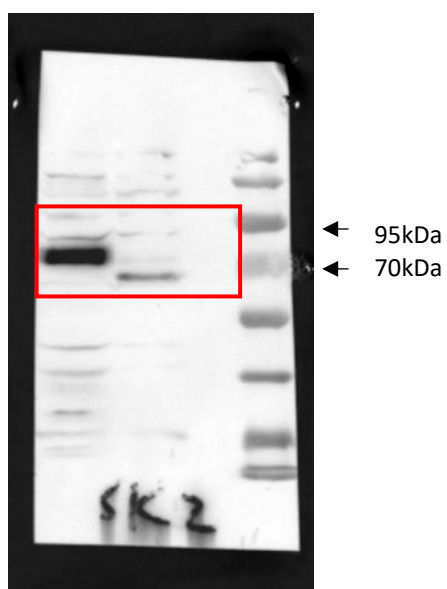

**$\beta$ -Actin**

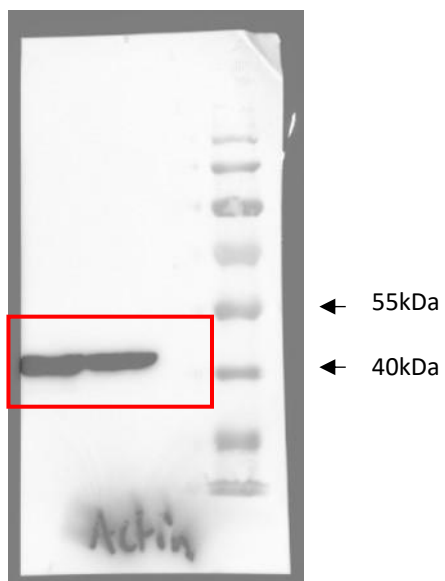

Supplement: Supplementary file 3 — Source Data for Expanded View and Appendix [file EMBJ-40-e106272-s006.zip › EV_and_Appendix_Source_Data/EMBOJ-2020-106272R1-Figure_EV3_Source_Data-sd.pdf]

Figure EV2C

P-P65

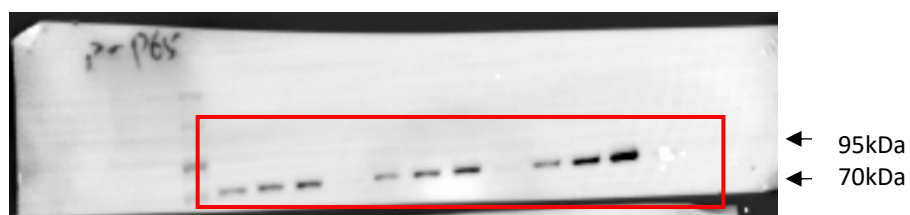

P-ERK

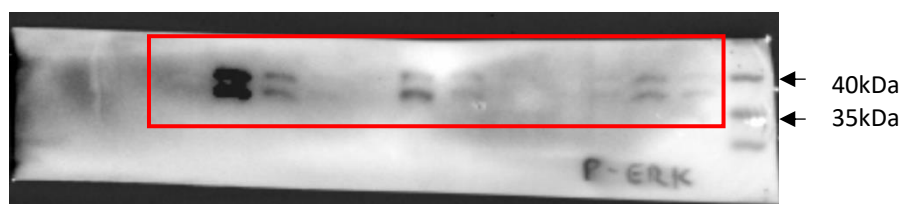

P-P38

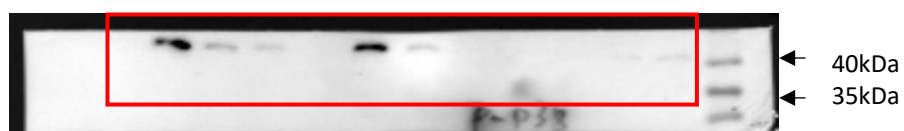

P-JNK

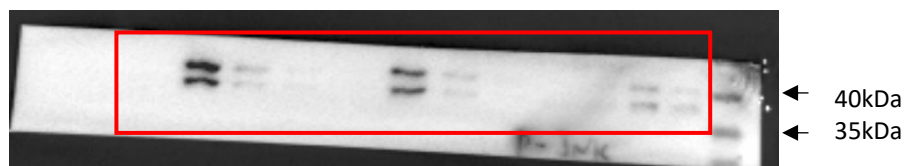

P65

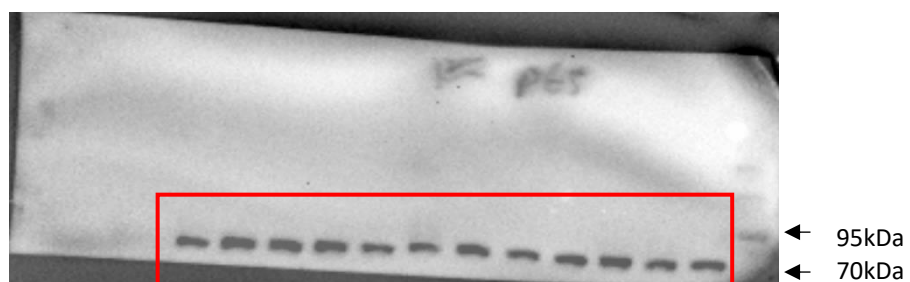

$\beta$ -Actin

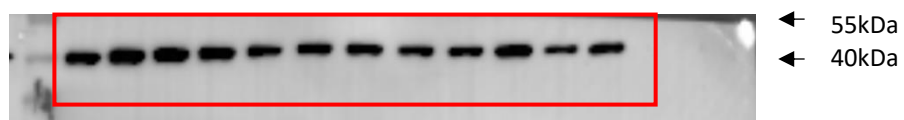

Supplement: Supplementary file 3 — Source Data for Expanded View and Appendix [file EMBJ-40-e106272-s006.zip › EV_and_Appendix_Source_Data/EMBOJ-2020-106272R1-Figure_EV2C_Source_Data-sd.pdf]
