# Supplementary material for: Cellular stress promotes NOD1/2‐dependent inflammation via the endogenous metabolite sphingosine‐1‐phosphate
Source: EMBO J. 2021 May 4;40(13):e106272. doi: 10.15252/embj.2020106272 (PMC8246065; doi:10.15252/embj.2020106272)

Figure 4C

IB: GFP (GFP IP)

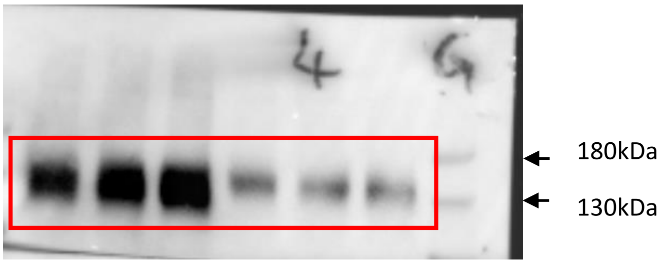

IB: GFP (Lysates)

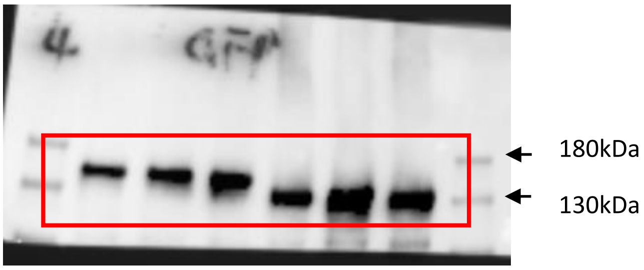

IB: RIP2 (GFP IP)

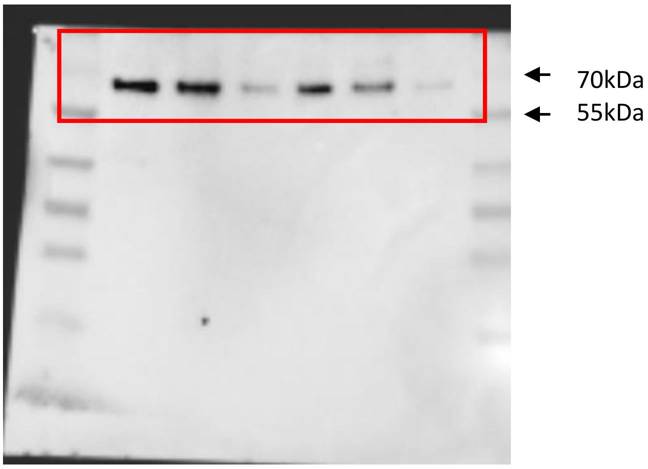

IB: RIP2 (Lysates)

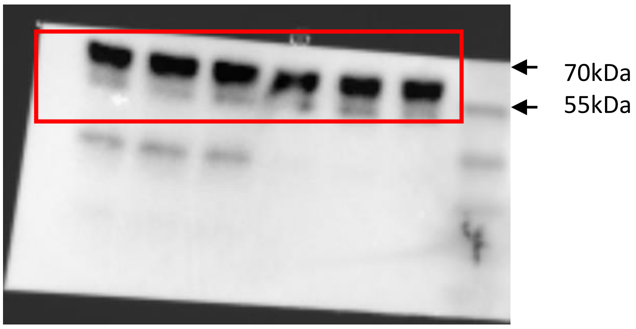

Figure 4D

P-JNK (Upper) and P-P38 (Lower)

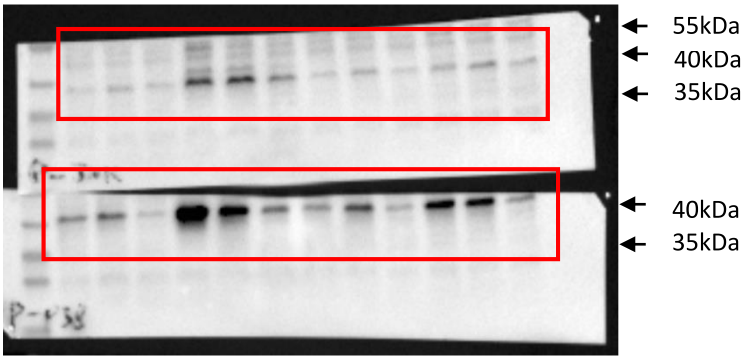

P-ERK

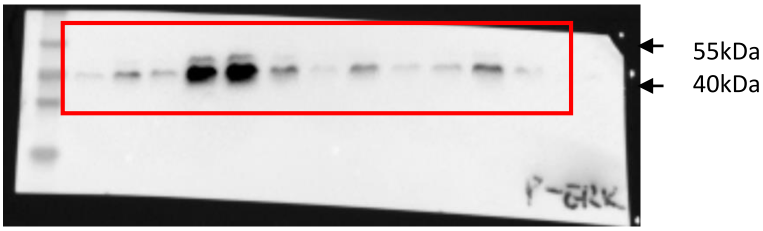

P-P65

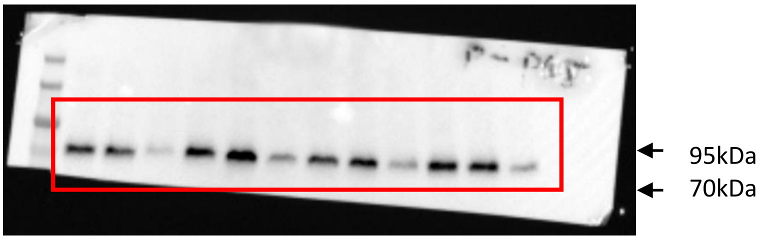

β-Actin (stripping after P-P38)

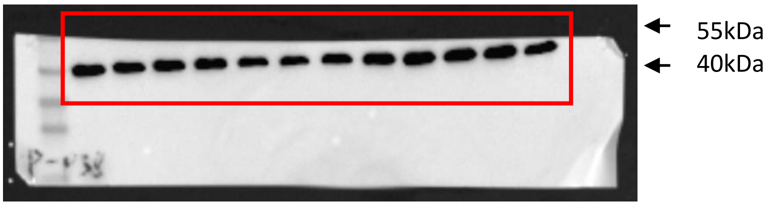

Supplement: Supplementary file 5 — Source Data for Figure 4 [file EMBJ-40-e106272-s005.pdf]
