# Supplementary material for: Cellular stress promotes NOD1/2‐dependent inflammation via the endogenous metabolite sphingosine‐1‐phosphate
Source: EMBO J. 2021 May 4;40(13):e106272. doi: 10.15252/embj.2020106272 (PMC8246065; doi:10.15252/embj.2020106272)

**Figure 5A**

**IB: GFP (NOD1-GFP, lipids-coated beads IP)**

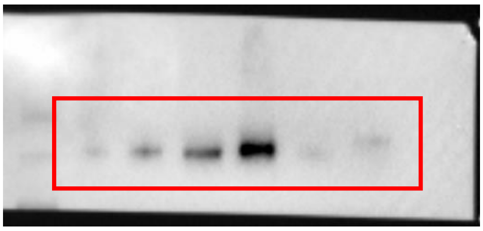

190kDa
130kDa

**IB: RIP2 (lipids-coated beads IP)**

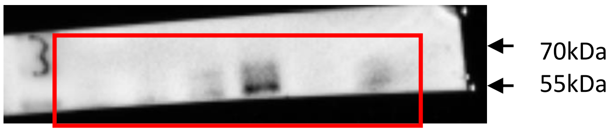

70kDa
55kDa

**IB: GFP (NOD1-GFP, Lysates)**

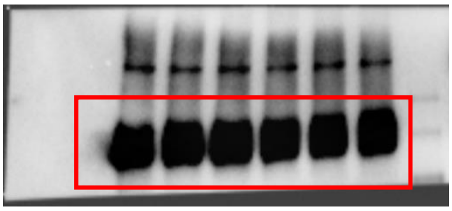

190kDa
130kDa

**IB: RIP2 (Lysates)**

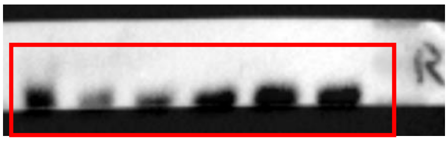

70kDa
55kDa

**Figure 5B**

**IB: GFP (NOD2-GFP, lipids-coated beads IP)**

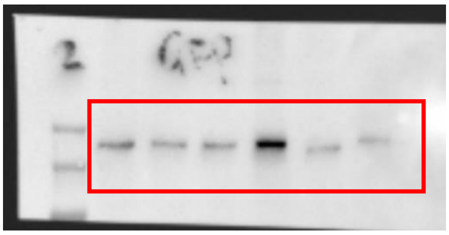

190kDa
130kDa

**IB: RIP2 (lipids-coated beads IP)**

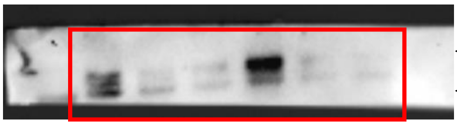

70kDa
55kDa

**IB: GFP (NOD2-GFP, Lysates)**

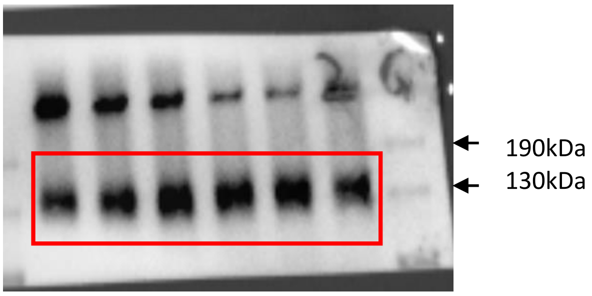


**IB: RIP2 (Lysates)**

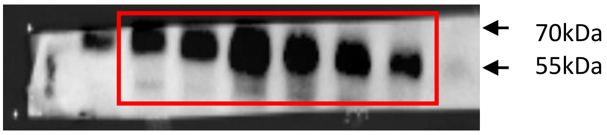


**Figure 5C**

**IB: NOD1**

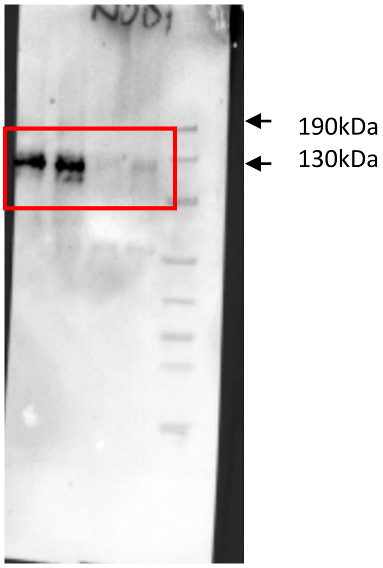

Supplement: Supplementary file 6 — Source Data for Figure 5 [file EMBJ-40-e106272-s002.pdf]
